# Residential Exposure to PM_2.5_ Components and Risk of Childhood Non-Hodgkin Lymphoma in Denmark: A Nationwide Register-Based Case-Control Study

**DOI:** 10.3390/ijerph17238949

**Published:** 2020-12-01

**Authors:** Ulla Arthur Hvidtfeldt, Friederike Erdmann, Stine Kjaer Urhoj, Jørgen Brandt, Camilla Geels, Matthias Ketzel, Lise M. Frohn, Jesper Heile Christensen, Mette Sørensen, Ole Raaschou-Nielsen

**Affiliations:** 1 Danish Cancer Society Research Center, Strandboulevarden 49, 2100 Copenhagen, Denmark; mettes@cancer.dk (M.S.); ole@cancer.dk (O.R.-N.); 2 Division of Childhood Cancer Epidemiology, Institute for Medical Biostatistics, Epidemiology and Informatics (IMBEI), Johannes Gutenberg University Mainz, Obere Zahlbacher Str. 69, 55131 Mainz, Germany; friederike.erdmann@uni-mainz.de; 3 Section of Epidemiology, Department of Public Health, University of Copenhagen, Oster Farimagsgade 5, P.O. Box 2099, DK-1014 Copenhagen K, Denmark; stur@sund.ku.dk; 4 Department of Environmental Science, Aarhus University, Frederiksborgvej 399, P.O. Box 358, 4000 Roskilde, Denmark; jbr@envs.au.dk (J.B.); cag@envs.au.dk (C.G.); mke@envs.au.dk (M.K.); lmf@envs.au.dk (L.M.F.); jc@envs.au.dk (J.H.C.); 5 Global Centre for Clean Air Research (GCARE), Department of Civil and Environmental Engineering, University of Surrey, Guildford GU2 7XH, UK; 6 Department of Natural Science and Environment, Roskilde University, Universitetsvej 1, P.O. Box 260, 4000 Roskilde, Denmark

**Keywords:** particulate matter components, Secondary organic aerosols, secondary inorganic aerosols, carbonaceous particles, childhood Non-Hodgkin Lymphoma, register-based study

## Abstract

In a recent study, we observed an increased risk of childhood non-Hodgkin lymphoma (NHL) associated with exposure to fine atmospheric particulate matter (PM_2.5_) and black carbon (BC). In this nationwide register-based case-control study, we focus on specific components of PM_2.5_ in relation to childhood NHL in Denmark (1981–2013) by identifying all incidents of childhood NHL cases in the Danish Cancer Registry (*n* = 170) and four (cancer-free) randomly selected controls matched by date of birth and sex. We applied PM_2.5_ concentrations and the following sub-components: secondary organic aerosols (SOA), secondary inorganic aerosols (SIA; i.e., NO_3_^−^, NH_4_^+^ and SO_4_^2−^), BC, organic carbon (OC) and sea salt. We calculated a time-weighted exposure average from birth to index-date at all addresses. Odds ratios (ORs) were adjusted for register-based socio-demographic variables. We observed adjusted ORs and 95% confidence intervals (95% CI) of 2.05 (1.10, 3.83) per interquartile range (IQR, 4.83 µg/m^3^) PM_2.5_ and 1.73 (0.68, 4.41) per IQR (3.71 µg/m^3^) SIA, 0.95 (0.71, 1.29) per IQR (0.05 µg/m^3^) SOA, 1.22 (1.02, 1.46) per IQR (0.39 µg/m^3^) BC, 1.02 (0.83, 1.26) per IQR (0.56 µg/m^3^) OC and 1.01 (0.79, 1.30) per IQR (0.87 µg/m3) sea salt, respectively. The estimates were attenuated after adjustment for PM_2.5_, whereas the OR for PM_2.5_ remained increased regardless of adjustment for specific components. The findings indicate that the previously observed relation between PM_2.5_ and childhood NHL may be related to BC (as reported in our previous study) but also partly to SIA, but the role of specific chemical components of PM_2.5_ remains ambiguous.

## 1. Introduction

Non-Hodgkin lymphomas (NHL) are a diverse group of malignancies of the immune system. Worldwide, NHL is the third most common type of childhood cancer (0–19 years) after leukemia and tumors of the central nervous system [1]. The age-standardized incidence rate varies considerably across regions with high rates observed in, for instance, specific European and African countries and lower rates in most parts of Asia [1].

The underlying causes of NHL are largely unknown; a male preponderance is observed and prior chemotherapy or medical radiation therapy as well as immunologic aspects, including having received an organ transplant or having suffered from Epstein-Barr, malaria and human immunedefiency virus infections, are suggested risk factors [2]. The observed rise in the incidence of childhood NHL over time combined with the geographical variation suggest an environmental component in the etiology of NHL. Maternal domestic pesticide exposure during pregnancy, exposure to ionizing radiation and parental smoking have been suggested as potential environmental risk factors [3,4]. Ambient air pollution is classified as a Group 1 carcinogen [5]; however, past studies have not reported clear evidence of an association with childhood NHL [6]. A recent Danish case-control study reported an association with maternal residential benzene exposure during pregnancy [7]. In a recently published study, we observed an association between residential exposure to ambient fine particulate matter (PM_2.5_) during childhood and the risk of NHL in Danish children with an observed odds ratio (OR) of 2.11 (95% confidence interval, CI: 1.10, 4.01) per 5 µg/m^3^ increments [8]. PM_2.5_, however, arises from various natural and anthropogenic sources and consists of a complex mixture of chemical components, of which some are released directly into the air (primary emitted) while others are formed by chemical reactions in the atmosphere (secondary). In the above-mentioned study, we also observed suggestive evidence of a role of the PM_2.5_ sub-component BC (black carbon). In two-pollutant models of PM_2.5_ mass and BC, an effect of PM_2.5_ remained, which indicates an influence of additional sub-components [8]. In order to prevent negative health effects of PM_2.5_, specific evidence on the relative influence of each chemical component is required. A previous study showed that carbonaceous particles (black/organic carbon, BC/OC) and secondary organic aerosols (SOA) mainly drove the association between PM_2.5_ exposure and NHL in Danish adults [9]. We have not been able to identify previous studies addressing the association between PM_2.5_ components (other than BC) and childhood NHL. 

In the present study, we elaborate on our previous findings by addressing the association between childhood NHL and primary emitted particles of BC, OC and sea salt and the secondary inorganic aerosols (SIA, i.e., NO_3_^−^, NH_4_^+^ and SO_4_^2−^, denoted as NO_3_, NH_4_ and SO_4_ in the following) and SOA in a population-based case-control study of the entire Danish population. The study includes all NHL cases among children (<20 years) in the period 1981–2013 linked to registered data, including individual residential address history, individual and neighborhood level socio-demographic information and a Danish high-resolution multiscale air pollution dispersion and human exposure modelling system.

## 2. Material and Methods

We conducted a nationwide matched case-control study based on linkage of individual data between Danish registries. From the Danish Cancer Registry [10], we identified all incident childhood NHL cases (aged 0–19 years) from 1 January 1981 to 31 December 2013 (*n* = 249). We defined NHL according to the International Classification of Childhood Cancer (i.e., the Birch and Marsden Classification and ICCC-3) by ICCC IIb [11,12]. We sampled four random controls matched to cases by sex and date of birth from the Danish Civil Registration System (CRS) [13] among children who were born in Denmark and who had no previous cancer diagnosis at the time of diagnosis of their matched case. The matching ensured identical follow-up times for cases and their matched controls.

Based on geocoded address histories obtained from the CRS for all cases and controls from 1979 onwards, we modelled front door (2 m height) PM_2.5_ concentrations and sub-components with the Danish modelling system DEHM/UBM/AirGIS [14,15,16]. Overall, the sub-components comprised the full PM_2.5_ mass, consisting of the primary emitted particles BC, OC, sea salt and mineral dust, and the additional secondary particle components SIA and SOA. The model is described in detail elsewhere [14,17]. In brief, three air pollution contributions are integrated into the system: (1) the regional background, modelled with the DEHM (Danish Eulerian Hemispheric Model) [18], covering the northern hemisphere, with higher resolution over Europe/Denmark (5.6 km × 5.6 km over Denmark). The DEHM includes all of the above mentioned PM_2.5_ components and processes the complex atmospheric chemistry which forms the SIA and SOA particles [19,20]. (2) The urban background, modelled with the UBM (Urban Background Model) [21], which handles the local contribution from primary anthropogenic sources of emitted particles (mineral dust, BC, OC) and calculates concentrations with a 1 km × 1 km resolution. (3) The local street level, modelled by the OSPM^®^ (Operational Street Pollution Model), which includes the type and intensity of traffic combined with emission factors, meteorology and street and building configuration [22]. We derived hourly concentrations of PM_2.5_ and sub-components from the system from 1979 onwards for all residential addresses. From these, a time-weighted average (TWA) concentration was calculated from birth to date of diagnosis or for controls from birth to date of diagnosis for their matched case (index-date).

We linked the study population to registers at Statistics Denmark [23,24], with information on parental education and disposable income one year before the index-date representing each child’s SES (socioeconomic status) during childhood. Disposable income was defined as the individual income after taxes, interests and alimony payments and we categorized this variable into deciles for mothers and fathers according to the sex- and calendar-year-specific income distribution of the general Danish population. We grouped the highest obtained educational level into basic (primary and lower secondary, ≤9 years), medium (upper secondary including vocational upper secondary, 10–12 years) and high education (>12 years), following the International Standard Classification of Education. We assessed information on parental age at birth of each child from the CRS and birth weight from the Medical Birth Register [25]. We assessed the number of siblings one year before the index-date, defined as all live-born siblings of either the same biological mother or father, from the Danish Fertility Database [26].

We obtained parish codes from the Danish Geodata Agency in order to define neighborhood SES, as described previously [17]. These data were available from 1986 onwards. We created three neighborhood-level SES measures based on the following: proportion of inhabitants in the age range 30–60 years with low disposable income (family disposable income among the lowest quartile of the income distribution of the entire Danish population), with only basic education and with a manual profession (unskilled or semi-skilled profession), respectively. We operationalized each neighborhood SES measure according to the quartiles of the distribution across all parishes in a given year weighted by the number of 30–60 year old inhabitants in the respective parish (i.e., five groups per measure). Information on neighborhood level SES was applied one year before the index-date.

We applied conditional logistic regression for exposure to PM_2.5_ and each sub-component separately in the following models: (1) a crude model (adjusted for age, sex and calendar time by matching) and (2) the main model which was further adjusted for individual-level covariates, i.e., parental age (included as a linear term), birth weight (categorized as <2500 g, 2500–3999 g and ≥4000 g), number of biological siblings (categorized as 0, 1, 2 or ≥3 siblings), parental education (basic, medium, high) and parental disposable income (linear). In analyses of a subsample of all cases occurring from 1987 onwards and corresponding controls, due to the limited availability of these data, we additionally added neighborhood SES covariates. We included exposures of PM_2.5_ and sub-components linearly per interquartile range (IQR) of their individual distribution. The test for deviation from linearity included a comparison of a decile model with a linear model using the likelihood ratio test. 

We performed the following sensitivity analyses: (1) Two-pollutant models to test whether the estimates of each sub-component were sensitive to adjustment for PM_2.5_ mass and vice versa. (2) Applying exposures during pregnancy, defined as the period from conception to birth according to gestational age at birth, adjusting for individual-level covariates measured at the time of conception. 

We excluded children for whom we were not able to geocode 80% or more address history in the period from birth to index-date (replacing up to 20% missing exposure by the TWA for the time with known exposure), children with missing information on individual-level covariates and consequently cases who did not have matching controls or vice versa following these exclusions (in total 79 cases and 387 controls). 

We performed all statistical analyses in SAS, version 9.4 (SAS Institute Inc., Cary, NC, USA) and R, version 3.6.3 (R-Core-Team, 2018).

## 3. Results

We included 170 NHL cases and 609 controls in the main model analyses. Table 1 and online Figure S1, respectively, show the levels of covariates according to case and control status and the exposure distribution of each sub-component of PM_2.5_. Overall, more than two-thirds of the study population were boys. Cases and controls did not differ considerably according to any of the considered covariates, except for minor differences in parental educational level, with a tendency towards less educated mothers and fathers among cases compared to controls. For the subsample of cases and controls with information on neighborhood SES, cases resided in a neighborhood with a high proportion of inhabitants with low income to a higher degree than controls, but cases were also more likely to live in a neighborhood with a low proportion of manual workers (Table 1). 

The crude and adjusted odds ratios (ORs) for exposure to PM_2.5_ and sub-components and risk of childhood NHL are presented in Table 2. In correspondence with our previous publication [8], the adjusted OR and 95% confidence interval (95% CI) per IQR of PM_2.5_ mass was 2.05 (1.10, 3.83) and 1.22 for BC (95% CI: 1.02, 1.46). We also observed an increased risk estimate for SIA (OR: 1.73, 95% CI: 0.68, 4.41), however with wide confidence intervals. Generally, adjusting for individual level covariates did not affect the effect estimates (Table 2). Adjusting for neighborhood level SES indicators attenuated, however, the OR for PM_2.5_ mass, SOA, BC and OC, and increased the effect estimate for SIA and sea salt (online Table S1).

The effect estimates for the association between sub-components of PM_2.5_ and NHL attenuated after adjustment for PM_2.5_ mass, except for sea salt for which the OR increased (Table 3). A slightly elevated risk estimate remained for BC and SIA with ORs of 1.12 (95% CI 0.87, 1.44) and 1.12 (95% CI 0.40, 3.17), respectively. The estimate for PM_2.5_ mass was attenuated following adjustment for BC and SIA and increased after adjustment for the three other sub-components, but remained elevated regardless of the component adjusted for.

Spearman correlation coefficients are presented in the online Table S2 and showed PM_2.5_ mass to be highly correlated with SIA and BC to be highly correlated with OC. BC and OC were moderately correlated with SOA and sea salt (negatively) whereas the remaining components were only mildly correlated. The power to perform two- and three-pollutant models in order to disentangle the separate effects of the separate SIA sub-components was limited due to high intercorrelations (Table S3) and resulted in very unstable results in two- and three-pollutant models (not shown).

In the sensitivity analyses, in which we applied exposure in the period from conception to birth, we observed somewhat lower effect estimates for PM_2.5_ mass, SIA, BC and sea salt compared to those of the main exposure analyses with adjusted ORs (95% CIs) of PM_2.5_ mass: 1.38 (0.87, 2.19) per IQR of 4.71 µg/m^3^; SIA: 1.21 (0.56, 2.60) per IQR of 3.64 µg/m^3^; BC: 1.12 (0.95, 1.31) per IQR of 0.45 µg/m^3^; and sea salt: 0.85 (0.66, 1.10) per IQR of 1.38 µg/m^3^. The effect estimate was higher for SOA and OC with an OR for SOA of 1.06 (0.80, 1.39) per IQR (0.14 µg/m^3^) and for OC of 1.16 (0.92, 1.46) per IQR (0.73 µg/m^3^).

## 4. Discussion

The findings of this study suggest that the possible role of PM_2.5_ in the development of childhood NHL could be partly related to BC, as suggested in our previous study, but possibly also related to SIA exposure.

To the best of our knowledge, our study is the first to explore the association between sub-components of PM_2.5_ and childhood NHL. We recently reported findings from a case-control study among the Danish adult population, which showed that carbonaceous particles (BC/OC) and secondary organic aerosols (SOA) mainly drove the association between PM_2.5_ exposure and adult NHL [9]. The literature on exposure to other groups of air pollutants and risk of childhood NHL is also sparse. In a case-control study from California, which explored traffic exposures during pregnancy and the first year of life, observed a slightly higher risk of NHL with higher residential traffic density and carbon monoxide exposure, but no such association for PM_2.5_ [27]. The study was, however, limited by a very low number of cases in the analysis of PM_2.5_ (*n* = 28). In a Canadian cohort study, a tendency towards a lower risk of NHL in children aged 0–5 years was observed with higher exposure to NO_2_ in pregnancy, but the study did not have the power to study the relationship with PM_2.5_ [28]. A recent large Swiss cohort study including more than 2 million children aged 0–15 years investigated the effect of traffic indicators at the residence in relation to lymphomas in general, but did not find support of such an association [29]. Furthermore, a previous Danish case-control study including cases from the period 1968–1991 reported an association between in utero exposure to NO_2_ and lymphomas [30]. In a later sub-analysis of this case-control sample, a positive association between benzene exposure during pregnancy and NHL was reported [7].

The proposed mechanisms by which PM_2.5_ may affect cancer risk include oxidative stress, systemic inflammation and epigenetic changes of the genome [5]. PM_2.5_ may plausibly affect the risk of NHL through the immune system, considering the markedly increased risks observed in adults who received organ transplants and immunosuppressive therapy and acquired immunodeficiency syndrome (AIDS) patients. The literature on the biological mechanisms by which different sub-components of PM_2.5_ may affect health is also sparse, but the mechanistic pathways likely vary. Both BC and SO_4_ have been found to lead to markers of systemic inflammation and SO_4_ and NH_4_ in PM_2.5_ have been linked to DNA methylation changes [31,32]. BC may also affect DNA methylation and induce oxidative stress [33,34].

The present study was strengthened by the relatively large sample size including all NHL cases occurring in children in Denmark over three decades and the application of nationwide registry data of high quality with nearly complete coverage and negligible risk of information- and non-participation bias [10]. The applied exposure modelling system DEHM/UBM/AirGIS combined with accurate residential address information from registries ensured high spatial and temporal precision in the exposure assignment. The PM_2.5_ levels in Denmark have declined over the past decades (Figure S2), however, the individual matching of cases and controls on calendar time ensured that identical calendar-time periods were compared in the analyses. We were able to address two different exposure time windows (childhood vs. pregnancy period) and adjust for a number of potentially important confounders such as parental age as well as education and income levels, the child’s birth weight, the number of siblings and neighborhood-level socio-demographic factors.

The high intercorrelations, especially between PM_2.5_ mass and SIA (*r_spearman_* = 0.917), as well as intercorrelations between SIA and the three SIA sub-components (*r_spearman_* > 0.9), challenged our ability to disentangle the relative importance of each component. Other limitations of this study include exposure misclassification following the application of a model for assessment of air pollution exposure; however, we assume the misclassification to be identical for cases and controls, which would lead to bias towards the null. Another challenge is that each modelled component might differ according to their level of uncertainty [14]. This could lead to a situation in which some of the causal effect is shifted from an inadequately modelled component to a more precisely modelled component [35,36,37]. The DEHM/UBM/AirGIS may underestimate some of the PM components in the DEHM, e.g., primary organic PM and secondary aerosols and non-exhaust PM from traffic in the OSPM such as tire wear, road wear and brake wear [14]. The applied version of DEHM/UBM/AirGIS only incorporated OC at the regional scale, which could vary from local OC due to uncertainties in wood stove PM emissions (e.g., number and position of woodstoves, wood use, emission factors and behavior of the user) [38]. Furthermore, we were not able to examine metal components of PM_2.5_ which play a significant role in the toxicity of PM_2.5_ by mechanisms of oxidative stress and inflammation [39]. We also did not have genetic information on the included children which could be an important factor for NHL risk. Future studies on the interaction between genes and environment could provide important new knowledge. Another point to consider is indoor sources of air pollution or exposures at other locations than at the children’s registered residential address. Lastly, we were not able to account for other environmental factors or lifestyle factors such as parental smoking, etc. We expect that the adjustment for socio-demographic factors accounted partly for these factors and note that the crude and adjusted estimates were close to identical in this study.

## 5. Conclusions

In conclusion, in addition to the previously reported influence of BC on childhood NHL development, this study suggests a role of SIA, but the findings need replication in other large-scale studies.

## Figures and Tables

**Table 1:** Characteristics of the study population, children (<20 years) with NHL (non-Hodgkin lymphoma) diagnosed 1981–2013 in Denmark and matched controls.

		Case-Control Status			
Characteristics		Cases		Controls	
		(*n* = 170)		(*n* = 609)	
**Sex**					
	Boys, *n* (%)	118	(69.4)	420	(69.0)
**Age group distribution**, *n* (%)					
	0–4 years	19	(11.2)	72	(11.8)
	5–9 years	51	(30.0)	187	(30.7)
	10–14 years	35	(20.6)	123	(20.2)
	15–19 years	65	(38.2)	227	(37.3)
**Birth weight**, *n* (%)					
	<2500 g	5	(2.9)	42	(6.9)
	2500–3999 g	131	(77.0)	443	(72.7)
	≥4000 g	34	(20.0)	124	(20.4)
**Maternal educational level**, *n* (%)					
	Basic (≤9 years)	38	(22.4)	99	(16.3)
	Medium (10–12 years)	76	(44.7)	303	(49.8)
	High (>12 years)	56	(32.9)	207	(34.0)
**Paternal educational level**, *n* (%)					
	Basic (≤9 years)	37	(21.8)	96	(15.8)
	Medium (10–12 years)	88	(51.8)	349	(57.3)
	High (>12 years)	45	(26.5)	164	(26.9)
**Maternal age**, mean (SD)		28.5	(4.9)	28.3	(4.9)
**Paternal age**, mean (SD)		31.1	(5.7)	31.0	(5.7)
**Maternal income**					
	Lowest quintile	6	(3.5)	24	(3.9)
	Highest quintile	68	(40.0)	234	(38.4)
**Paternal income**					
	Lowest quintile	11	(6.5)	48	(7.9)
	Highest quintile	52	(30.6)	193	(31.7)
**Siblings**, *n* (%)					
	0	16	(9.4)	60	(9.9)
	1	83	(48.8)	297	(48.8)
	2	46	(27.1)	162	(26.6)
	3+	25	(14.7)	90	(14.8)
**Neighborhood SES**, *n* (%)		(*n* = 165)		(*n* = 594)	
*PI with basic education*					
	Lowest quintile	56	(33.9)	205	(34.5)
	Highest quintile	14	(8.5)	65	(10.9)
*PI with low income*					
	Lowest quintile	51	(30.9)	186	(31.3)
	Highest quintile	27	(16.4)	62	(10.4)
*PI with manual*					
	Lowest quintile	56	(33.9)	156	(26.3)
	High quintile	24	(14.6)	89	(15.0)

Abbreviations: NHL, non-Hodgkin lymphoma; SES, socioeconomic status; PI, proportion inhabitants.

**Table 2:** Associations between PM2.5 components from birth to index-date and risk of childhood NHL diagnosed in Denmark 1981–2013(cases/controls 170/609).

Pollutant	IQR	OR (95% CI)	
		Crude ^a^	Adjusted ^b^
PM_2.5_	4.83	2.09 (1.14, 3.84)	2.05 (1.10, 3.83)
SIA	3.71	1.75 (0.71, 4.33)	1.73 (0.68, 4.41)
SOA	0.05	0.98 (0.74, 1.31)	0.95 (0.71, 1.29)
BC	0.39	1.22 (1.03, 1.45)	1.22 (1.02, 1.46)
OC	0.56	1.04 (0.85, 1.26)	1.02 (0.83, 1.26)
Sea salt	0.87	1.00 (0.78, 1.27)	1.01 (0.79, 1.30)

^a^ Adjusted (by matching) for age, sex and calendar year; ^b^ Further adjusted for parental age, birth weight, number of biological siblings, parental education and parental disposable income; Abbreviations: NHL, non-Hodgkin lymphoma; PM, particulate matter; SIA, secondary inorganic aerosols; SOA, secondary organic aerosols; BC/OC, black/organic carbon.

**Table 3:** Two-pollutant models of the association between PM_2.5_ and PM_2.5_ components from birth to index-date and risk of childhood NHL diagnosed in Denmark 1981–2013.

		OR (95% CI)		
Pollutant	IQR	Single-Pollutant ^a^	Sub-Components Adjusted for PM_2.5_^a^	PM_2.5_ Adjusted for Sub-Components ^a^
PM_2.5_	4.83	2.05 (1.10, 3.83)	-	-
SIA	3.71	1.73 (0.68, 4.41)	1.12 (0.40, 3.17)	1.99 (1.01, 3.94)
SOA	0.05	0.95 (0.71, 1.29)	0.80 (0.57, 1.12)	2.45 (1.24, 4.82)
BC	0.39	1.22 (1.02, 1.46)	1.12 (0.87, 1.44)	1.57 (0.65, 3.75)
OC	0.56	1.02 (0.83, 1.26)	0.90 (0.71, 1.14)	2.33 (1.17, 4.64)
Sea salt	0.87	1.01 (0.79, 1.30)	1.14 (0.87, 1.50)	2.31 (1.19, 4.48)

^a^ Adjusted for age (by matching), sex (by matching), calendar year (by matching), parental age, birth weight, number of biological siblings, parental education and parental disposable income. Abbreviations: NHL, non-Hodgkin lymphoma; PM, particulate matter; SIA, secondary inorganic aerosols; SOA, secondary organic aerosols; BC/OC, black/organic carbon.

## Supplementary Materials

The following are available online at https://www.mdpi.com/1660-4601/17/23/8949/s1, Figure S1: Exposure distributions of each sub-component of PM_2.5_ among controls (*n* = 609) for the period from birth to index-date (µg/m^3^), Figure S2: Population-weighted mean PM_2.5_ (µg/m^3^) concentrations over Denmark for the period 1979–2019, calculated with the DEHM/UBM model system on a 1 km × 1 km resolution. Table S1: Associations between PM_2.5_ components from birth to index-date and risk of NHL diagnosed in Denmark 1987–2013 with further adjustment for area level socioeconomic variables among the sub-population with this information, Table S2: Spearman correlation coefficients between PM_2.5_ mass and sub-components from birth to index-date among controls, Table S3: Spearman correlation coefficients between SIA particles and the three sub-components from birth to index-date among controls.
