# Supplementary material for: Residential Exposure to PM2.5 Components and Risk of Childhood Non-Hodgkin Lymphoma in Denmark: A Nationwide Register-Based Case-Control Study
Source: Int J Environ Res Public Health. 2020 Dec 1;17(23):8949. doi: 10.3390/ijerph17238949 (PMC7729659; doi:10.3390/ijerph17238949)
Supplement: Supplementary file 1 [file ijerph-17-08949-s001.pdf]

Supplementary

Hvidtfeldt, UA et al. Residential exposure to $PM_{2.5}$ components and risk of childhood non-Hodgkin Lymphoma in Denmark: A nationwide register-based case-control study

Figure S1 Exposure distributions of each sub component of $PM_{2.5}$ among controls (N=609) for the period from birth to index-date (µg/m^3)

Figure S2 Population weighted mean $PM_{2.5}$ (µg/m^3) concentrations over Denmark for the period 1979-2019, calculated with the DEHM/UBM model system on a 1 km x 1 km resolution.

Table S1 Associations between $PM_{2.5}$ components from birth to index-date and risk of NHL diagnosed in Denmark 1987–2013 with further adjustment for area level socioeconomic variables among the sub-population with this information

Table S2 Spearman correlation coefficients between $PM_{2.5}$ mass and sub components from birth to index-date among controls

Table S3 Spearman correlation coefficients between SIA particles and the three sub components from birth to index-date among controls

Figure S1. Exposure distributions of each sub component of $PM_{2.5}$ among controls (N=609) for the period from birth to index-date ($\mu g/m^3$).

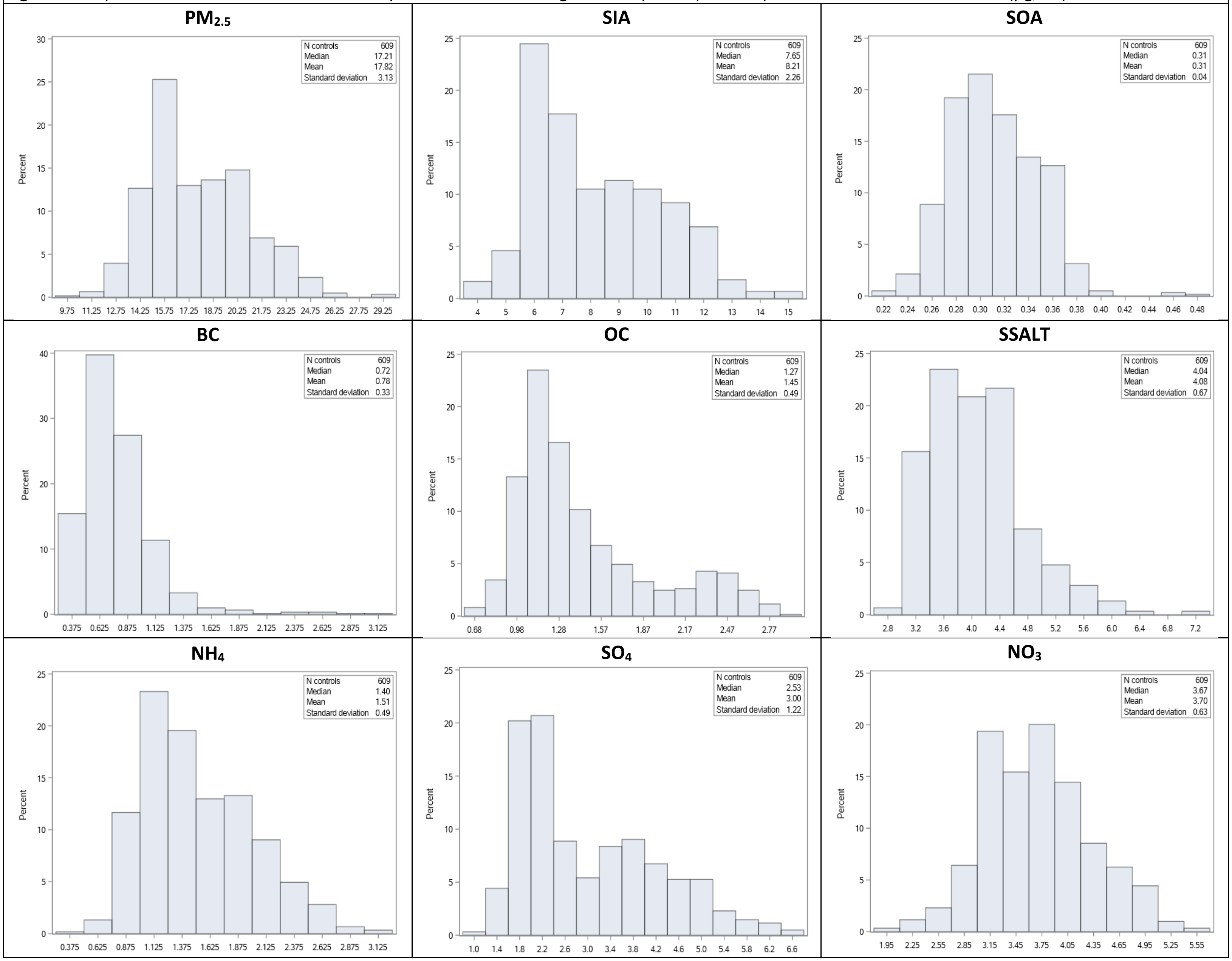

Figure S2. Population weighted mean $PM_{2.5}$ ($\mu g/m^3$) concentrations over Denmark for the period 1979-2019, calculated with the DEHM/UBM model system* on a 1 km x 1 km resolution

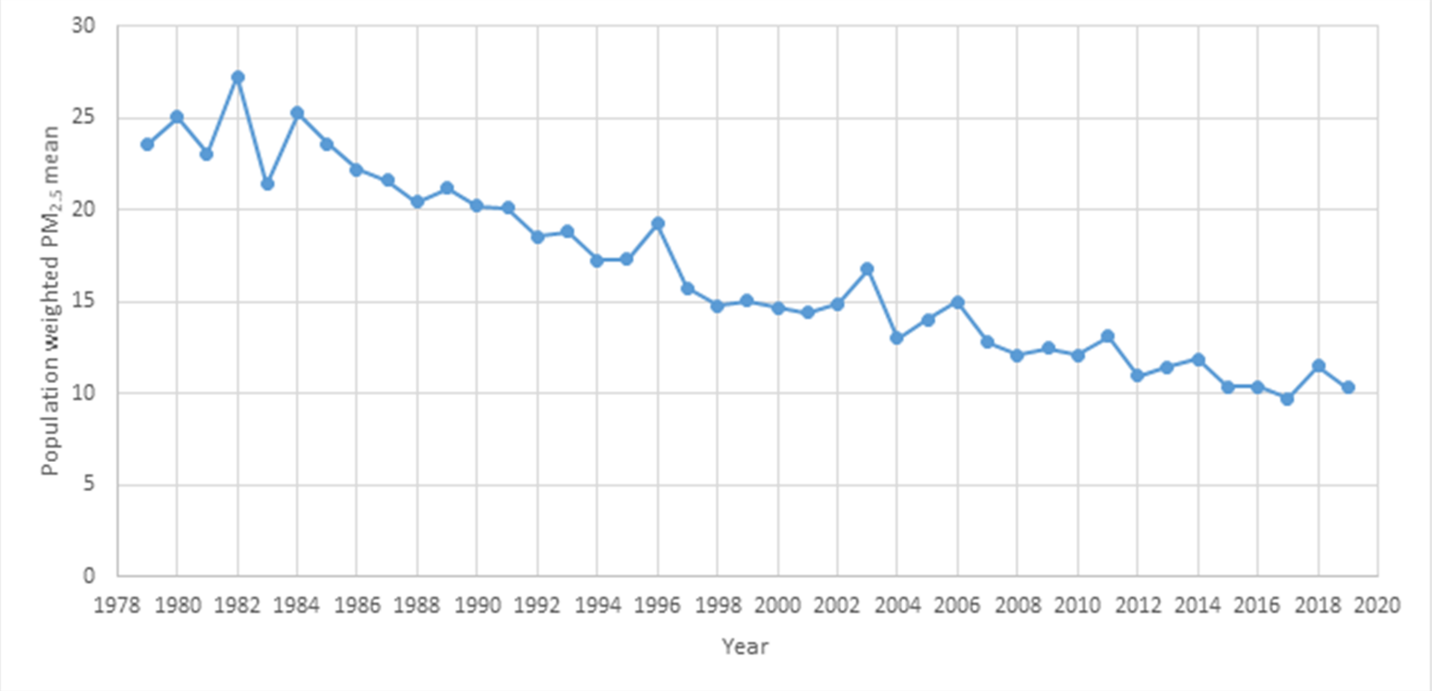

*Brandt, J.; Christensen, J.H.; Frohn, L.M.; Berkowicz, R. Air pollution forecasting from regional to urban street scale -implementation and validation for two cities in Denmark. *Phys. Chem. Earth, Parts A/B/C* **2003**, *28*, 335–344, doi:10.1016/S1474-7065(03)00054-8.

Table S1. Associations between $PM_{2.5}$ components from birth to index-date and risk of NHL diagnosed in Denmark 1987–2013 with further adjustment for area level socioeconomic variables among the sub-population with this information (cases/controls 165/594).

		OR (95% CI)	
**Pollutant**	**IQR**	**Adjusted individual level**[a]	**Adjusted + neighborhood SES**
$PM_{2.5}$	4.83	2.16 (1.13, 4.12)	2.04 (1.03, 4.06)
SIA	3.71	1.64 (0.63, 4.29)	1.84 (0.66, 5.14)
SOA	0.05	1.02 (0.75, 1.39)	0.86 (0.60, 1.24)
BC	0.39	1.25 (1.04, 1.50)	1.23 (1.00, 1.52)
OC	0.56	1.05 (0.85, 1.29)	0.90 (0.70, 1.16)
Sea salt	0.87	0.97 (0.75, 1.25)	1.07 (0.80, 1.43)

[a]Adjusted for age (by matching), sex (by matching), calendar year (by matching), parental age, birth weight, number of biological siblings, parental education, and parental disposable income.

Abbreviations: NHL, non-Hodgkin lymphoma; PM, particulate matter; SIA, secondary inorganic aerosols; SOA, secondary organic aerosols; BC/OC, black/organic carbon

Table S2. Spearman correlation coefficients between $PM_{2.5}$ mass and sub components from birth to index-date among controls (N = 609).

	**$PM_{2.5}$**	**SIA**	**SOA**	**BC**	**OC**
**SIA**	0.917	-	-	-	-
**SOA**	-0.022	-0.239	-	-	-
**BC**	0.454	0.147	0.560	-	-
**OC**	0.295	0.019	0.746	0.822	-
**Sea salt**	0.067	0.257	-0.831	-0.608	-0.672

Abbreviations: PM, particulate matter; SIA, secondary inorganic aerosols; SOA, secondary organic aerosols; BC/OC, black/organic carbon

Table S3. Spearman correlation coefficients between SIA particles and the three sub components from birth to index-date among controls (N = 609).

	**SIA**	**NO_3**	**NH_4**
**NO_3**	0.965	-	-
**NH_4**	0.970	0.945	-
**SO_4**	0.979	0.905	0.922

Abbreviations: SIA, secondary inorganic aerosols
